# Antimicrobial resistance temporal trend of Klebsiella pneumoniae isolated from blood

**Published:** 2016

**Authors:** LC Gavriliu, OE Benea, S Benea

**Affiliations:** * Carol Davila” University of Medicine and Pharmacy, Bucharest, Romania; “Prof. Dr. Matei Bals” National Institute of Infectious Diseases, Bucharest, Romania

**Keywords:** Klebsiella pneumoniae, antimicrobial resistance

## Abstract

**Background.** According to the European Antimicrobial Resistance Surveillance Network, Romania reports an increasing number of resistant Klebsiella pneumoniae strains from invasive infections every year.

**Material and Method.** We analyzed the antimicrobial susceptibility of Klebsiella pneumoniae strains isolated from blood in 2010 and 2015 in “Matei Bals” National Institute of Infectious Diseases, in order to identify any significant changes in the last five years.

**Results.** We identified 18 strains in 2010 and 37 strains in 2015. Although the resistance to aminopenicillin-betalactamase inhibitors association, piperacillin-tazobactam, third generation cephalosporins, fluoroquinolones, gentamicin, amikacin and the combined resistance decreased between these two time frames, this evolution was statistically non-significant. The same was noticed for the increased resistance rates to carbapenems.

**Conclusions.** Antimicrobial resistance of Klebsiella pneumoniae may become a major problem for the public health and the hospital-acquired infections control. Therefore, it needs further monitoring and efforts must be made in order to limit the increase of the resistance.

## Background

Klebsiella pneumoniae (K. pneumoniae) is a clinical and epidemiological significant bacteria involved in infectious diseases. K. pneumoniae is associated with bacteremia (second place among gram-negative rods after E. coli), urinary and respiratory tract infections [**1**]. 

In Eastern, Southern and Central Europe there is a high rate of multi-drug resistant K. pneumoniae. Among these countries, Romania reports an increasing number of resistant K. pneumoniae strains isolated from invasive infections every year. The most recent report of our country to the European Antimicrobial Resistance Surveillance Network (EARS-Net) has placed us as among the first places regarding K. pneumoniae resistance to fluoroquinolones (third place after Slovakia and Greece), third generation cephalosporins (second place after Bulgaria), aminoglycosides (second place after Slovakia), carbapenems (third place after Italy and Greece), as well as its multidrug resistance (third place after Slovakia and Greece) [**1**].

The rates of antimicrobial resistance are not homogenous throughout different regions of the world. Therefore, the antimicrobial treatment guidelines for a specific geographic area must be based upon local antimicrobial resistance data. It is important for clinicians to know these resistance data in order to be able to make the best antibiotic treatment choice.

## Material and Method

We conducted a non-interventional, retrospective study in “Prof. Dr. Matei Bals” National Institute of Infectious Diseases in Bucharest, Romania. The purpose of this study was to analyze K. pneumoniae from blood susceptibility to antimicrobials and to compare the resistance rates between these two periods in order to identify any statistically significant changes during a five years time frame. The analyzed periods were January 1st – December 31st 2010 and January 1st – December 31st 2015. We used real-life data provided by the microbiology laboratory. The data wereusedas they werecommunicated toclinicians, without making any additional considerationsabout resistance profiles. 

After excluding the bacterial duplicates (the same strain isolated from a patient in less than 4 weeks), the antibiograms of all the remaining 55 isolates from blood were studied (18 isolates in 2010 and 35 isolates in 2015). We analyzed the resistance to the antibiotics considered relevant for epidemiological surveillance by EARS-Net [**1**] and WHO [**2**] and also those that the authors have considered relevant for their clinical experience. The antimicrobial susceptibility tests were performed by semi-automated methods (API, Vitek, Microscan). The statistical analysisof the data wasperformed with EPIINFO software 3.4.3. The statistical significance was assessed by calculating the statistical significance threshold (p value) using Fisher test. P was considered significant for a value less than 0.05.

## Results

We analyzed 55 K. pneumoniae isolates from blood: 18 identified in 2010 (T1) and 35 identified in 2015 (T2).

Among the tested antibiotics included in the testing panels for gram-negative rods, the following were considered clinically and epidemiologically relevant: aminopenicillins, aminopenicillin-betalactamaseinhibitor association, piperacillin-tazobactam, 3rd generation cephalosporins, fluoroquinolones, aminoglycosides, and carbapenems. 

Not all of the isolated strains were tested to all the antibiotics. The lowest testing rates in 2010 were identified for aminopenicillin-betalactamase inhibitor associations, third generation cephalosporins, and piperacillin-tazobactam. All of them improved in 2015 (**Table 1**).

**Table 1:** Klebsiella pneumonia strains isolated from blood testing, resistance and its evolution

Antimicrobial	Tested T1	Resistance T1	Tested T2	Resistance T2	Evolution of the resistance T1-T2 (p)
Aminopenicillins	17/ 18 (94.44%)	16/ 17 (94.11%)	35/ 37 (94.59%)	35/ 35 (100%)	↑ (p=0.32)
Aminopenicillin-betalactamase inhibitor	13/ 18 (72.22%)	6/ 13 (46.15%)	36/ 37 (97.29%)	13/ 36 (36.11%)	↓ (p=0.52)
Piperacillin - Tazobactam	16/ 18 (88.88%)	6/ 16 (37.5%)	37/ 37 (100%)	9/ 37 (24.32%)	↓ (p=0.34)
3rd generation cephalosporins	13/ 18 (72.22%)	5/ 13 (38.46%)	37/ 37 (100%)	11/ 37 (29.72%)	↓ (p=0.73)
Carbapenems	17/ 18 (94.44%)	0/ 17 (0%)	37/ 37 (100%)	1/ 37 (2.7%)	↑ (p=1)
Fluoroquinolones	17/ 18 (94.44%)	8/ 17 (47.06%)	37/ 37 (100%)	10/ 37 (27.02%)	↓ (p=0.21)
Aminoglycosides	17/ 18 (94.44%)	7/ 17 (41.17%)	37/ 37 (100%)	8/ 37 (21.62%)	↓ (p=0.19)
ESBL	18/ 18 (100%)	4/ 18 (22.22%)	37/ 37 (100%)	11/ 26 (29.73%)	↑ (p=0.74)
MDR	18/ 18 (100%)	7/ 18 (38.88%)	37/ 37 (100%)	8/ 37 (21.62%)	↑ (p=0.20)
*Legend: Tested T1 = number of strains tested in 2010 (percentage), Resistance T1 = number of resistant strains in 2010 (percentage), Tested T2 = number of strains tested in 2015 (percentage), Resistance T2 = number of resistant strains in 2015 (percentage), Evolution of the resistance T1-T2 = evolution of the resistance between 2010-2015, ↑ = increasing resistance, ↓ = decreasing resistance, p = statistical value (statistically significant at a value below 0.05), MDR = multidrug resistant, ESBL = extended-spectrum betalactamase producing strains*.					

**Fig. 1:**
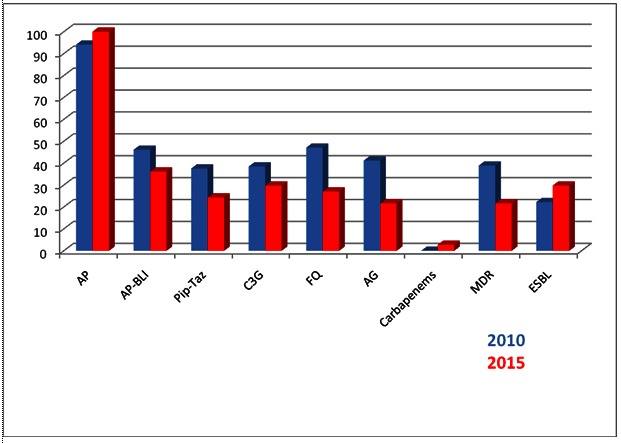
Resistance rates of Klebsiella pneumonia strains isolated from blood in 2010 and 2015 Legend: AP = aminopenicillins, AP-BLI = aminopenicillin-betalactamase inhibitor, Pip-Taz = piperacillin-tazobactam, C3G = third generation cephalosporins, FQ = fluoroquinolones, AG = aminoglycosides, MDR = multidrug resistant, ESBL = extended-spectrum betalactamase producing strains.

Among the 18 strains of K. pneumoniae isolated from blood in 2010 the resistance rates were 94.11% to aminopenicillin, 46.15% to aminopenicillin-betalactamase inhibitor associations, 37.7% to piperacillin-tazobactam, 38.46% to third generation cephalosporins, 47.07% to fluoroquinolones, 41.17% to aminoglycosides. We did not find any strains resistant to carbapenems in 2010. In 38.88% of the strains, we identified a combined resistance, meaning that the isolates were resistant to at least three classes of antimicrobials from fluoroquinolones, third generation cephalosporins, aminoglycosides, and carbapenems. Because no carbapenem resistance was found at that time, the combined resistance applied for the first three classes. 22.22% of the strains were extended-spectrum betalactamases (ESBL) producing (**Table 1**, **Fig. 1**).

In 2015, 37 strains of K. pneumoniae were isolated from blood. Among them, the resistance rates were 100% to aminopenicillins, 36.11% to aminopenicillin-betalactamase inhibitors association, 24.32% to piperacillin-tazobactam, 29.72% to third generation cephalosporins, 27.02% to fluoroquinolones, 21.62% to aminoglycosides. One strain was resistant to carbapenems. We found 21.62% of the strains to have a combined resistance and 29.73% ESBL (**Table 1**, **Fig. 1**).

When we compared the difference in efficiency of aminopenicillin-betalactamase inhibitors association, piperacillin-tazobactam, and third generation cephalosporins against K. pneumoniae, we found no statistically significant differences in the activity of these antimicrobials for any one of the two analyzed time frames (**Table 2**).

**Table 2:** The efficiency of aminopenicillin-betalactamase inhibitors association vs. piperacillin-tazobactam vs. third generation cephalosporins against Klebsiella pneumonia isolates from blood

Antimicrobial/ year	2010	2015
Aminopenicillin-betalactamase inhibitors association	p = 0.71	p = 0.31
Piperacillin-Tazobactam	p = 1	p = 0.62
Third generation cephalosporin	p = 1	p = 0.79
*Legend: p = statistical value (statistically significant at a value below 0.05)*.		

Analyzing the evolutionary trend of antimicrobial resistance rates between 2010 and 2015, we found an increasing resistance to aminopenicillins, aminopenicillin-betalactamase inhibitor associations, and carbapenems. We also found an increasing rate of ESBL strains. For all the other classes of the tested antimicrobials, as well as for the combined resistance, we noticed a decreasing tendency. However, none of these evolutions had any statistical significance (**Table 1**, **Fig. 1**).

We also analyzed the background of the patients who provided the isolates and compared it against the strains that displayed the most important resistant aspect (ESBL, multidrug resistance, resistance to carbapenems). For 2010, 4 patients were transferred from other hospitals, 12 patients were admitted from home (but two of them had previous hospitalizations in the previous six months) and for 2 patients it was not specified. In the same year, all the ESBL strains came from the patients who were transferred, and the combined resistant strains came from the four transferred patients, the two patients with previous hospitalization and one patient who was admitted from home. For 2015, 8 patients were transferred from other healthcare facilities, 22 patients were admitted from home (5 of them being hospitalized in the last six months), 5 patients had unspecified hospitalization history. All of the 8 multidrug resistant strains were obtained from patients who were transferred. The 11 ESBL strains were obtained from the 8 transferred patients and 3 of those previously hospitalized. The carbapenems resistant strain was obtained from a transferred patient. 

## Discussions

The temporal antimicrobial trend analysis showed an increasing rate of Enterobacteriaceae resistance to all antibiotic classes. K. pneumoniae is one of the bacteria that displayed this tendency, along with E. Coli [**3**].

Aminopenicillins cannot be used as a treatment option for the infections caused by K. pneumoniae, because this bacterium is intrinsically resistant to this antibiotics class as it harbors chromosomally encoded class Abetalactamase [**1**]. The association of a betalactamase inhibitor partially reestablishes the efficiency of aminopenicillins. Even in this case, the resistance rates remain high and eliminate this association from the first line empiric treatment options for these infections (46.16% in 2010 and statistically non-significant decrease to 36.11% in 2015). 

The resistance rate of K. pneumoniae to piperacillin-tazobactam is high (37.5% in 2010 with a statistically non-significant decrease to 24.36% in 2015). Higher resistance rates to these antimicrobials (96.9%) were reported by other authors, but in case of ESBL producing isolates [**4**].

K. pneumoniae resistance to third generation cephalosporins was very close to the previous one for each of the analyzed periods and with the same statistically non-significant decreasing pattern (38.46% in 2010 and 29.72% in 2015). The finding in our study is different from the data reported by our country to EARS-Net: increasing resistance from 44% in 2011 to 73.8% in 2014, when we occupied the second place after Bulgaria [**1**]. In our study, we demonstrated the presence of ESBL for 22.22% of the strains tested to cephalosporins in 2010 and 29.73%, but the rate of the confirmatory tests was low. This shows an undervaluation of this problem.

We did not find any differences between the efficiency of aminopenicillinsbetalactamase inhibitor associations, piperacillin-tazobactam, and third generation cephalosporins against K. pneumoniae, although in the first case, the resistance rate was higher. According to our findings, any of these antibiotics are an equally reasonable choice for the treatment of K. pneumoniae bacteremia.

We found a high rate of resistance to aminoglycosides in 2010 (41.17%) but with an important improvement, although statistically non-significant in 2015 (21.62%). If in 2011, Romania reported to EARS-Net a resistance rate of K. pneumoniae to aminoglycosides somewhat closer to our findings (50%), in 2014 it increased to 67.3%, both the percentage and the evolutionary trend being different from our study [**1**]. One explanation for this, could be the fact that in our study, the antimicrobial resistance testing was sometimes performed to alternative antibiotics from the same class (for example: gentamicin-amikacin), that could alter the accuracy of our data.

A very high resistance rate of K. pneumoniae to fluoroquinolones was noted in 2010 (47.06%) and the fact that it decreased in 2015 (27.02%) could be a sign that the plea of the infectious diseases specialists for fluoroquinolones consumption reduction has been heard and applied. However, our results are discordant with those from EARS-Net, where an increase of the resistance rate was noted, from 30% in 2010 to 66.5% in 2014 [**1**]. High rates of resistance were also found (40.4% Madagascar) in other regions of the globe [**5**].

The carbapenem resistance is a concerning growing problem all over the world. If in 2011 our country reported to EARS-Net 10 isolates of K. pneumonia resistant to carbapenems [**6**], in 2014 we reported 81 (31.5%) [**1**]. In our study, the carbapenems’ activity was excellent against K. pneumoniae strains isolated from blood (no resistance in 2010 and 1 resistant strain 2015). However, the presence of the carbapenem resistance among these isolates could be a major problem for the public health and hospital-acquired infections control. Therefore, it requires the reassessment of these strains and the CMI evaluation. The phenomenon of carbapenemase-producing K. pneumoniae is already spreading all around Europe and the situation has deteriorated in the last years, according to EuSCAPE - the “European survey of carbapenemase-producing Enterobacteriaceae” project launched in 2012 by the European Centre for Disease Prevention and Control [**6**]. In Romania, the first confirmed cases of OXA-48 and NDM-1 producing Enterobacteriaceae, mostly K. pneumoniae, were isolated in 2012 and both of them were predominant until 2013 [**7**-**9**].

The increase in multidrug resistance, along with the increase percentage of ESBL is a serious problem all over the world and it led to an increased use of carbapenems. Consecutively, the spread of carbapenemase-producing bacteria will be favored and the clinicians will be left with limited therapeutic options for these infections. According to EARS-Net, our country occupies the third place in Europe regarding the multidrug resistance of K. pneumoniae isolated from invasive infections. In our study, the multidrug resistant rate decreased between 2010 (38.88%) and 2015 (21.26%), again discordant from the data reported to EARS-Net (30% in 2011 and 56% in 2014). 

There were some limitations in our study. Some problems related to susceptibility tests may have influenced the results: limited number of isolates, alternative tests with substances from the same class of antibiotics (imipenem-meropenem, ciprofloxacin-levofloxacin, and ceftriaxone-cefotaxime), the lack of the tests for some antibiotics, insufficient verification of some results that indicated unexpected resistance. Therefore, although the decreasing resistance rates that we observed are hopeful, they must be considered with caution, and further monitoring of this pathogen antimicrobial resistance is mandatory. 

## Conclusions

1. The percentage of K. pneumoniae strains isolated from blood increased between 2010 and 2015.

2. Between 2010 and 2015 we did not find any statistically significant changes of the resistance rates of K. pneumoniae to all the classes of tested antimicrobials, although the decreasing resistance rates for all the analyzed antimicrobials were observed. 

3. We found one strain resistant to carbapenems in 2015.The presence of the carbapenem resistance among these isolates could be a major problem for the public health and hospital-acquired infections control.

4. Our data regarding the proportion of resistant K. pneumoniaestrains isolated from blood are different from those reported by our country to EARS-Net. We found lower and decreasing resistance rates to fluoroquinolones, aminoglycosides, third generation cephalosporins, carbapenems, and combined resistance. Still, with Romania occupying ones of the first places in Europe regarding K. pneumoniae isolated from invasive infections resistance to antibiotics, further monitoring is mandatory and efforts should be made in order to limit this problem.

## References

[1] www.ecdc.europa.eu. Antimicrobial resistance surveillance in Europe 2014.

[2] WHO/CDS/CSR/DRS/2001.2. WHO global strategy for containment of antimicrobial resistance 2001.

[3] Rhomberg PR, Jones RN (2009). Summary trends for Meropenem Yearly Susceptibility Test Information Program: a 10-year experience in the United States (1999-2008). Diagnostic Microbiology and Infectious Diseases.

[4] Maina D, Makau P, Nyerere A, Revathi G (2013). Antimicrobial resistance patterns in extended spectrum betalactamase producing E. Coli and Klebsiella pneumoniae isolates in a private tertiary hospital, Kenia. Microbiology Discovery.

[5] Randrianirina F, Vaillant L, Ramarokoto CE, Rakotoarijaona A, Andriamanarivo ML, Razafimahandry HC, Randrianomenjanahary J, Raveloson JR, Hariniana ER, Carod JF, Talarmin A, Richard V (2010). Antimicrobial resistance in pathogens causing nosocomial infections in surgery and intensive care wards in Antananarivo, Madagascar. J Infect Dev Ctries.

[6] Albiger B, Glasner C, Struelens M, Grundmann H, Monnet D (2015). The European Survey of Carbapenemase-Producing Enterobacteriaceae (EuSCAPE) working group. Carbapenemase-producing Enterobacteriaceae in Europe: assessment by national experts from 38 countries, May 2015. Euro Surveill.

[7] Székely E, Damjanova I, Jánvári L, Vas KE, Molnar S, Bilca DV, Löriczi LK, Tóth A (2013). First description of bla(NDM-1), bla(OXA-48), bla(OXA-181) producing Enterobacteriaceae strains in Romania. Int J Med Microbiol.

[8] Dortet L, Flonta M, Boudehen YM, Creton E, Bernabeu S, Vogel A, Naas T (2015). Dissemination of Carbapenemase-Producing Enterobacteriaceae and Pseudomonas aeruginosa in Romania. Antimicrob Agents Chemother.

[9] Gheorghe I, Czobor I, Chifiriuc MC, Borcan E, Ghita C, Banu O, Lazar V, Mihaescu G, Mihailescu DF, Zhiyong Z (2014). Molecular screening of carbapenemase-producing Gram-negative strains in Romanian intensive care units during a one year survey. J Med Microbiol.

